# GABA-producing *Lactococcus lactis* alleviates gut dysfunction and neurobehavioral abnormalities associated with irritable bowel syndrome

**DOI:** 10.20517/mrr.2025.56

**Published:** 2025-09-23

**Authors:** Zhiying Jin, Mengyu Chen, Lanxi Ao, Jingyu Wang, Jingge Sun, Xin Qian, Peijun Tian, Hao Zhang

**Affiliations:** ^1^State Key Laboratory of Food Science and Resources, Jiangnan University, Wuxi 214122, Jiangsu, China.; ^2^School of Food Science and Technology, Jiangnan University, Wuxi 214122, Jiangsu, China.; ^3^National Engineering Research Center for Functional Food, Jiangnan University, Wuxi 214122, Jiangsu, China.; ^4^(Yangzhou) Institution of Food Biotechnology, Jiangnan University, Yangzhou 225004, Jiangsu, China.

**Keywords:** IBS, GABA, *Lactococcus lactis*, gut-brain axis

## Abstract

**Aim:** This study aimed to screen *Lactococcus lactis* strains with varying gamma-aminobutyric acid (GABA) production and evaluate their effects on intestinal dysfunction and neurobehavioral abnormalities in an irritable bowel syndrome (IBS) mouse model, with a focus on GABAergic signaling and dose-dependent mechanisms.

**Methods:** Three *Lactococcus lactis* strains were selected based on GABA yield and genetic analysis. IBS was induced in mice via *Citrobacter rodentium* infection and water avoidance stress. Intestinal integrity, inflammation, histopathology, and behavior were assessed. GABA levels in the colon and serum were measured by liquid chromatography-mass spectrometry (LC-MS). GABA receptor subunit expression in the colon, hippocampus, and amygdala was analyzed via quantitative real-time polymerase chain reaction and Western blotting.

**Results:** GABA-producing strains alleviated intestinal dysfunction in IBS mice by reducing *IL-6* gene expression and iNOS activity, upregulating *CLDN2*, and improving tissue integrity. Anxiety-like behaviors and cognitive deficits were also attenuated. Colonic GABA levels, *GABRA13* mRNA, and GABRA3 protein expression increased in a dose-dependent manner, whereas TRPV1 mRNA and TRPV1 protein levels were downregulated. Serum GABA remained unchanged. In the central nervous system, the expression of hippocampal GABA_A_ and GABA_B_ receptors was elevated, with both *GABRA13* mRNA and GABRA3 protein levels positively correlating with colonic GABA concentrations. *GABRA15* expression was upregulated in the amygdala.

**Conclusion:** GABA-producing *Lactococcus lactis* effectively alleviates IBS-related intestinal dysfunction and neurobehavioral abnormalities by coordinately modulating GABAergic signaling in both the gut and the central nervous system, exhibiting a clear dose-dependent effect across multiple key phenotypes.

## INTRODUCTION

Irritable bowel syndrome (IBS) is a chronic functional gastrointestinal disorder characterized by recurrent abdominal pain and altered bowel habits, with highly heterogeneous clinical manifestations and a complex etiological basis^[1]^. According to the internationally recognized Rome IV criteria, IBS is classified into four subtypes^[2]^, among which the diarrhea-predominant subtype (IBS-D) is the most prevalent^[3]^, significantly impairing patients' quality of life. Current clinical treatments primarily aim to relieve symptoms, including the use of antispasmodics and antidiarrheal agents, or to manage psychological comorbidities through the administration of anxiolytic and antidepressant medications^[4]^. However, these approaches often demonstrate limited efficacy and are associated with high relapse rates, posing challenges for sustained long-term management^[5]^. The pathophysiology of IBS is multifactorial, involving genetic susceptibility, dietary habits, psychological stress, intestinal infections, immune dysregulation, and dysfunction of the gut-brain axis^[6]^. Among these, impaired gut-brain communication has been widely acknowledged as a central mechanism underlying IBS pathogenesis. Emerging evidence suggests that IBS patients frequently exhibit neurobehavioral abnormalities, including visceral hypersensitivity, dysregulated central sensory processing, and comorbid anxiety and depression. These findings indicate that IBS is not merely a gastrointestinal disorder, but also involves aberrant central processing of peripheral gut-derived signals^[7]^.

In recent years, the gut microbiota - recognized as a key regulatory component of the gut-brain axis - has garnered increasing attention for its role in IBS. Emerging evidence indicates that individuals with IBS commonly exhibit alterations in both the composition and metabolic profiles of the gut microbiome. Compared to healthy controls, IBS patients display reduced overall microbial diversity, an increased abundance of specific pathogenic taxa (e.g., *Ruminococcus*, *Clostridium sensu stricto*), and a decreased prevalence of anti-inflammatory genera such as *Bifidobacterium* and *Faecalibacterium*^[8,9]^. A matched cohort study from the American Gut Project (*n* = 942 IBS *vs.* 942 healthy controls) further revealed significant alterations in microbial functional metabolism in IBS patients, including disruptions in short-chain fatty acid (SCFA) production and glutamate-Gamma-aminobutyric acid (GABA) metabolic pathways^[10,11]^.

GABA is a neuroactive metabolite synthesized and secreted by various gut bacteria, such as *Lactobacillus* and *Bifidobacterium*^[12]^. It is not only the major inhibitory neurotransmitter in the central nervous system (CNS), but also a key signaling molecule in the enteric nervous system (ENS), where it regulates gastrointestinal motility, secretion, and immune responses. Previous studies have shown that supplementation with GABA or the intake of GABA-producing probiotics can alleviate emotional and sensory disturbances such as anxiety, depression, and pain^[13]^. Additionally, gut-derived GABA can activate GABA receptors in the ENS, thereby modulating intestinal motility, reducing neuronal sensitivity, and exerting anti-inflammatory effects^[14]^, making it a potential therapeutic target for IBS. However, oral GABA is limited by poor blood-brain barrier permeability^[15]^ and rapid absorption in the proximal intestine, restricting its availability to colonic and local immune targets^[16]^. In contrast, GABA-producing probiotics can synthesize GABA *in situ*, enabling localized and sustained release within the gut, thereby more effectively modulating ENS activity, reducing neuronal sensitivity, and exerting anti-inflammatory effects^[17,18]^. Moreover, probiotics provide additional benefits such as strengthening barrier function and maintaining immune homeostasis, offering synergistic effects beyond GABA alone^[19,20]^. These features make probiotic-based strategies a natural, safe, and sustainable alternative to pharmacological GABA supplementation, with strong potential for long-term use in functional foods and clinical nutrition.

Among the various GABA-producing bacteria, *Lactococcus lactis* has attracted attention due to its Generally Recognized As Safe (GRAS) status and the high GABA synthesis capacity observed in certain strains^[21]^. Compared to other lactic acid bacteria such as *Lactiplantibacillus plantarum* and *Lacticaseibacillus rhamnosus*, which have been extensively studied^[22]^, *Lactococcus lactis* is primarily used in dairy fermentation, and its biological functions - particularly in terms of metabolite production and neuromodulatory activity - remain relatively underexplored^[23]^. Currently, studies investigating the regulatory role of GABA-producing *Lactococcus lactis* strains via the gut-brain axis are still limited, and the underlying mechanisms require further elucidation.

In this study, we aimed to: (1) screen representative *Lactococcus lactis* strains with varying GABA production capacities; (2) evaluate their effects and underlying mechanisms in alleviating symptoms in a mouse model of IBS; and (3) assess whether these effects exhibit dose dependency, thereby providing theoretical support and new therapeutic insights for microbiota-based interventions in IBS.

## METHODS

### Bacteria and culture conditions

*Lactococcus lactis* strains used in this study were originally isolated from healthy human feces or fermented foods and are preserved in the Culture Collections of Food Microbiology in Jiangnan University (CCFM, Wuxi, China). The specific species and sources are listed in Table 1. Strains stored in glycerol stocks were thawed and a small amount of culture was streaked onto MRS agar plates using an inoculating loop. Plates were incubated at 37 °C for 48 h. A single colony was then selected and inoculated into 5 mL of liquid MRS broth for further cultivation. The second-generation culture was used as the seed inoculum for subsequent fermentation to accumulate GABA.

**Table 1:** *Lactococcus lactis* strain information

**Number**	**Species**	**Origins**	**Area**
CICC6246	*Lactococcus lactis*	Purchased from CICC	Jiangsu, China
s15m9	*Lactococcus lactis*	Dairy products	Neimenggu, China
5G2	*Lactococcus lactis*	Fermentation samples	Yunnan, China
M137R01G2	*Lactococcus lactis*	Adult feces	Henan, China
8（ZN8)	*Lactococcus lactis*	Pickle	Jiangsu, China
DXJ412	*Lactococcus lactis*	Sour mare's milk	Neimenggu, China
DYNDL195	*Lactococcus lactis*	Dairy fan	Neimenggu, China
DYNDL61M6	*Lactococcus lactis*	Dairy fan	Neimenggu, China
M2143	*Lactococcus lactis*	Qula	Sichuan, China
HeNa218GMM	*Lactococcus lactis*	Dairy products	Jiangsu, China
DQHXNQ05M30	*Lactococcus lactis*	Qula	Qinghai, China
DSCAB2M1	*Lactococcus lactis*	Dairy products	Neimenggu, China
DSCAB4M9	*Lactococcus lactis*	Dairy products	Neimenggu, China
DSCAB12M2	*Lactococcus lactis*	Dairy products	Yunnan, China
FSDHZD1L1	*Lactococcus lactis*	Dairy products	Yunnan, China
FSDHZD8L3	*Lactococcus lactis*	Dairy products	Yunnan, China
VNMWLT1M5	*Lactococcus lactis*	Vegetables	Hainan, China
FBJ3T3	*Lactococcus lactis*	Feces	Hainan, China
N5	*Lactococcus lactis*	Chicken manure	Hainan, China
6G5	*Lactococcus lactis*	Dairy products	Sichuan, China
HeNa283GMM	*Lactococcus lactis*	Feces	Sichuan, China
DYNDL343	*Lactococcus lactis*	Dairy products	Sichuan, China
H30G6	*Lactococcus lactis*	Feces	Hubei, China

*Citrobacter rodentium* (ATCC® 51459^TM^ DBS100) was thawed from glycerol stock and streaked onto LB agar plates using a sterile inoculating loop. Plates were incubated at 37 °C for 48 h. A single colony was then inoculated into 5 mL of LB and grown to logarithmic phase. Subsequently, 2% v/v of this culture was transferred into 500 mL of LB and incubated until mid-log phase. Bacterial cells were harvested by centrifugation and resuspended at the desired concentration for use in animal experiments.

### Qualitative identification of GABA-producing *Lactococcus lactis* strains using thin-layer chromatography

*Lactococcus lactis* cultures were grown in MRS broth supplemented with 1% (w/v) monosodium glutamate (MSG) at 37 °C for 48 h. Following incubation, cultures were centrifuged at 12,000 × *g* for 10 min, and the resulting supernatants were collected for further analysis. Aliquots of the supernatants were spotted onto silica gel 60 GF254 thin-layer chromatography (TLC) plates (50 mm × 200 mm; Macklin Biochemical Co., Ltd., Cat# S829510-1EA). Chromatographic separation was performed using a mobile phase consisting of n-butanol: acetic acid: water (5:3:2, v/v/v). After development, the plates were air-dried and subsequently sprayed with 0.5% (w/v) ninhydrin dissolved in ethanol as the chromogenic reagent. GABA was identified by the appearance of a characteristic purple spot, confirmed by comparison with a GABA reference standard (TargetMol, Cat# T0508) applied in parallel. The retention factor (Rf) and color intensity were used as indicators to verify the presence of GABA in the tested samples.

### Quantification of GABA by high-performance liquid chromatography

For the determination of GABA concentration, high-performance liquid chromatography (HPLC) was conducted using a Thermo Scientific system equipped with an ACQUITY BEH C18 column (1.7 µm, 2.1 mm × 100 mm; Waters, USA). The column temperature was maintained at 30 °C, with a flow rate of 0.2 mL/min and an injection volume of 5 µL. Ultraviolet detection was performed at 338 nm. The mobile phase consisted of 20 mmol/L sodium acetate in Milli-Q water (A) and a 1:1 (v/v) mixture of 40 mmol/L sodium acetate and HPLC-grade acetonitrile (B). The gradient elution procedure was programmed as follows: 0-6 min, 30%-50% B; 6-11 min, 50%-60% B; 11-15 min, 60%-100% B; 15-16 min, 100%-30% B; and 16-20 min, 30% B. Prior to injection, samples underwent pre-column derivatization with o-phthalaldehyde (OPA; TargetMol, CAS No. 643-79-8). The reagent was prepared by dissolving 0.1 g OPA in 1 mL of HPLC-grade acetonitrile, adding 130 µL β-mercaptoethanol, and diluting to 100 mL with freshly prepared 0.4 mol/L borate buffer (pH 10.2). A volume of 10 µL of the derivatization reagent was mixed with 10 µL of the filtered supernatant and reacted at room temperature for 90 s before injection.

### Bioinformatic analysis

*Lactococcus lactis* strains collected from the Culture Collections of Food Microbiology in Jiangnan University, along with type strains retrieved from the NCBI database, were subjected to whole-genome comparative analysis. Homologous genes were clustered, and core/pan-genome profiling was conducted using the Roary pipeline. A phylogenetic tree was constructed from core gene alignments using the approximate maximum-likelihood method and visualized with interactive tree of life.

Genomic similarity among strains was assessed using FastANI to compute average nucleotide identity (ANI), and results were displayed as a heatmap.

To explore candidate enzymes associated with GABA synthesis, hidden Markov model (HMM) domain scanning was performed. Pfam domain PF00282 (GadB) and TIGRFAM entry TIGR00910.1 (GadC) were used as queries. HMMER v3.0 was applied to scan the proteomes of all strains to identify proteins containing these conserved domains.

### Animal experiment design

All procedures were approved by the Ethics Committee of Jiangnan University (JN. No20240630c0500910[327]). Male C57BL/6 mice (10 weeks old) were maintained under SPF conditions. After a 7-day acclimatization period, mice were randomly assigned to 5 groups (*n* = 8 per group): control + stress (CS), model + stress (MS), H-GABA (DYNDL61M6), L-GABA (FSDHZ_D1_L1), and No-GABA (M2_14_3). Except for the CS group, all mice were infected orally with *Citrobacter rodentium* (10^9^ CFU/day) on Day 7. From Day 8 to 37, mice received daily gavage with 10^8^ CFU/day of the probiotics; CS and MS groups received saline. To induce stress, water avoidance stress (WAS) was performed daily from Days 25 to 37. Behavioral tests (open field, novel object recognition, and forced swim) were carried out from Days 38 to 40.

### Behavioral tests

The open field test (OFT) was conducted first to evaluate exploratory behavior and anxiety-like responses. Each mouse was placed in the center of a 40 cm × 40 cm × 40 cm open-field arena and allowed to explore freely for 6 min. Behavior was recorded using an automated video-tracking system, and parameters such as total distance traveled, total time spent moving, and time spent in the center zone were analyzed.

The novel object recognition (NOR) test was performed to assess recognition memory. The protocol consisted of 3 phases: habituation (free exploration in the empty arena for 5-10 min), familiarization (exploration of 2 identical objects for 5 min), and testing (1 familiar object replaced with a novel object for 3-5 min). Discrimination and recognition indices were calculated to evaluate memory performance.

**Figure:**
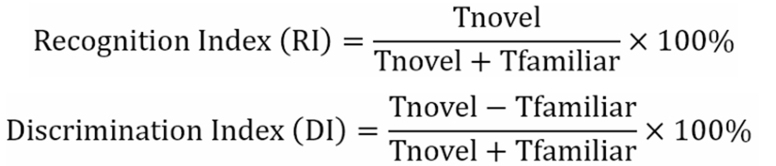


The forced swim test (FST) was performed to assess depression-like behavior. Mice were placed individually into transparent cylinders filled with water (depth: 15-20 cm; temperature: 23-25 °C). A 10-min pre-test session was performed on Day 1, followed by a 5-min test session on Day 2. The duration of immobility (defined as floating with minimal movements to keep the head above water) was recorded as an indicator of behavioral despair.

### Measurement of inducible nitric oxide synthase activity in colon tissue

The activity of inducible nitric oxide synthase (iNOS) in colon tissue was determined using a Nitric Oxide Synthase (NOS) Assay Kit (Cat# A014-1-2; Jiancheng Bioengineering Institute, Nanjing, Jiangsu, China). The assay is based on the enzymatic reaction of L-arginine with molecular oxygen to produce nitric oxide (NO), which subsequently reacts with a nucleophilic reagent to generate a chromogenic product. The absorbance was measured at 530 nm, and iNOS activity was calculated accordingly. Results were expressed as units of iNOS activity (U iNOS) per milligram of colon tissue protein.

### Colonic hematoxylin and eosin staining and crypt depth measurement

To evaluate the severity of colonic inflammation in mice, hematoxylin and eosin (H&E) staining was performed following standard histological procedures^[24]^. Images were captured using a digital slide scanner. Morphometric analysis was conducted using Image-Pro Plus 6.0 software. For each section, five representative fields were selected, and the crypt depth, mucosal thickness, muscularis thickness, and total wall thickness were measured. The mean values were calculated and used for statistical analysis.

### Determination of fecal moisture content

At the conclusion of the gavage intervention (Day 37), mice were individually housed in clean cages lined with absorbent paper to facilitate fecal collection. Freshly excreted fecal samples were immediately weighed to obtain the wet weight. The samples were then lyophilized to constant weight to determine the dry weight. Fecal moisture content was calculated using the following equation:

**Figure:**
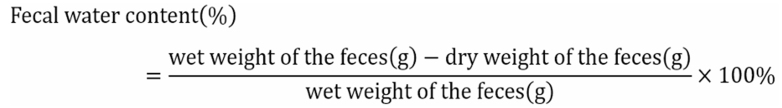


### Quantification of GABA in fecal and serum samples by liquid chromatography-mass spectrometry

Approximately 20 mg of fecal samples were homogenized with zirconium beads (30 mg) at 50 Hz for 10 min, followed by centrifugation at 12,000 × *g* for 10 min. Serum samples (40 μL) were incubated at -20 °C for protein precipitation, then centrifuged at 15,000 × *g* for 10 min. Supernatants (120 μL) from both sample types were vacuum-dried and reconstituted in 60 μL of 98:2 water/acetonitrile for liquid chromatography-mass spectrometry (LC-MS) analysis.

Metabolites were separated on a HILIC column (2.1 mm × 150 mm, 1.7 μm) at 35 °C, using a binary mobile phase: Solvent A (10 mm ammonium formate in 50:50 acetonitrile/water) and Solvent B (10 mm ammonium formate in 15:5:80 water/methanol/acetonitrile), with a flow rate of 0.3 mL/min and 20 μL injection volume. Gradient elution was conducted over 11 min, and the autosampler was kept at 4 °C.

### Quantitative real-time polymerase chain reaction

Total RNA was extracted from mouse colon, hypothalamus, and striatum tissues using the TRIzol reagent (Life Technologies, USA). Approximately 20-30 mg of tissue was placed in 1 mL of TRIzol along with 2 RNase-free zirconium beads and thoroughly homogenized. RNA was then purified according to the manufacturer’s protocol, involving chloroform extraction, isopropanol precipitation, and 75% ethanol washing. RNA concentration and purity were measured spectrophotometrically.

Reverse transcription was performed using a commercial kit (HiScript® III RT SuperMix for quantitative polymerase chain reaction (qPCR), Cat# R333-01, Vazyme Biotech Co., Ltd., Nanjing, China). Briefly, 1 μg of total RNA was reverse transcribed in a 20 μL reaction volume under the following conditions: 50 °C for 8 min, 85 °C for 5 s, followed by 10 °C for 30 s. The resulting cDNA was diluted 1:4 with DEPC-treated water and used as a template for quantitative real-time PCR(RT-qPCR). Gene-specific primers used for amplification are listed in Table 2.

**Table 2:** Sequence of the primers

**Gene**	**Primer sequence (5’-3’)**
*β-actin*	CCTTCCAGCAGATGTGGATCA
	CTCAGTAACAGTCCGCCTAGAA
*IL-6*	TGTGACTCCAGCTTATCTCTTGG
	GACAAAGCCAGAGTCCTTCAGA
*IL-10*	GCTCTTACTGACTGGCATGAG
	CGCAGCTCTAGGAGCATGTG
*CLDN2*	ATGCCTTCTTGAGCCTGCTT
	AAGGCCTAGGATGTAGCCCA
*GABRA11*	AAAAGCGTGGTTCCAGAAAA
	GCTGGTTGCTGTAGGAGCAT
*GABRA12*	GCTACGCTTACACAACCTCAGA
	GACTGGCCCAGCAAATCATACT
*GABRA13*	GCCGTCTGTTATGCCTTTGTATTT
	TTCTTCATCTCCAGGGCCTCT
*GABRA14*	AGAACTCAAAGGACGAGAAATTGT
	TTCACTTCTGTAACAGGACCCC
*GABRA15*	GATTGTGTTCCCCATCTTGTTTGGC
	TTACTTTGGAGAGGTGGCCCCTTTT
*GABBR1*	TGGTTTCTCATCGGGTGGTAT
	CCAAGGCCCAGATAGCATCA
*GABBR2*	ACATGCAAAGACCCCATAGAG
	TCGTGAGAGTAAGACCGTCG
*TRPV1*	CCTGGTGGTGGTCTTCATCT
	TGGCATAGACGGTGATGATG

### Western blot analysis

Protein expression levels were determined using a fully automated capillary-based Western blot system (ProteinSimple Wes, Model PS-MK15; ProteinSimple, USA). Total protein concentrations from mouse colon and brain tissue lysates were first quantified using the bicinchoninic acid (BCA) assay. Subsequently, the samples were loaded into dedicated capillary cartridges designed for the system.

The entire process - including protein separation, transfer, blocking, antibody incubation, washing, and chemiluminescent detection - was carried out automatically within the instrument. Target proteins included GABRA11 (Cat# BD-PT5569, Biodragon, Beijing, China) and GABRA13 (Cat# BD-PB3944, Biodragon, Beijing, China), TRPV1 (Cat# BD-PB3944, Biodragon, Beijing, China), and GAPDH (Cat# AC001, ABclonal, Wuhan, China). GAPDH was diluted 1:2,000, and all other primary antibodies were diluted 1:100 using the manufacturer’s provided antibody diluent. All procedures were performed according to the manufacturer’s instructions using a 13-lane assay plate. Protein signal acquisition and semi-quantitative analysis were conducted using the system’s dedicated software.

### Data analysis

All data are expressed as mean ± standard deviation (mean ± SD). Statistical analyses and graphical visualizations were performed using GraphPad Prism 9.0 (GraphPad Software, USA). One-way analysis of variance (ANOVA) was used to compare the CS group with the MS group and the intervention groups. For comparison between the two groups, an unpaired *t*-test was applied using GraphPad Prism (version 10.0). Significance levels are denoted as follows: *P < 0.05, **P < 0.01, ***P < 0.001, and ****P < 0.0001.

## RESULTS

### Phylogenetic analysis of key GABA biosynthesis genes and screening of high-GABA-producing Lactococcus lactis strains

**Figure 1:**
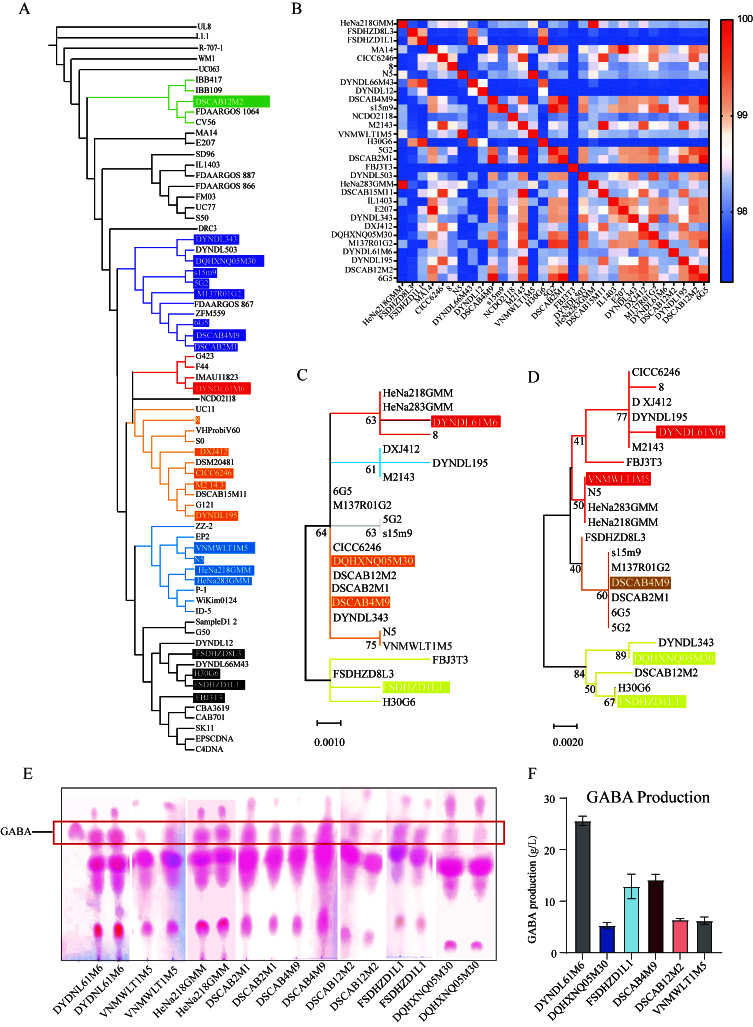
Screening of representative GABA-producing *Lactococcus lactis* strains. (A) A phylogenetic tree of *Lactococcus lactis* strains was constructed using the approximately-maximum-likelihood method. Strains from the Culture Collections of Food Microbiology at Jiangnan University are indicated in different colors, highlighting their positions and evolutionary branches within the tree; (B) ANI among *Lactococcus lactis* strains in the strain collection; (C and D) Phylogenetic analysis of *GadB* and *GadC* gene sequences in *Lactococcus lactis* strains from Jiangnan University; (C) Phylogenetic tree based on *GadB* sequences; (D) phylogenetic tree based on *GadC* sequences. Colored branches indicate the clustering and distribution of GABA-producing strains; (E) Qualitative assessment of GABA production by *Lactococcus lactis* strains using TLC; (F) Quantitative determination of GABA production in TLC-identified positive strains using ANI. GABA: Gamma-aminobutyric acid; TLC: thin-layer chromatography; HPLC: high-performance liquid chromatography; ANI: average nucleotide identity.

To elucidate the genetic background of *Lactococcus lactis* strains in our strain collection, we integrated genomes of strains preserved at Jiangnan University with publicly available genomes from NCBI. A phylogenetic tree was constructed based on core genome sequences, and ANI values were calculated [Figure 1A and B]**.** The results showed that all strains clustered into 6 major evolutionary branches, with ANI values exceeding 95%, indicating high genomic homology. Notably, strains DSCAB12M2 and DYDNL61M6 formed distinct sub-branches within the phylogenetic tree, exhibiting marked genetic divergence. Strain FBJ3T3 also displayed considerable ANI variation. Subsequently, we performed phylogenetic analysis targeting key enzyme genes involved in the GABA biosynthetic pathway [Figure 1C and D]. These genes were highly conserved among strains and could be classified into two major functional clades.

Preliminary screening of GABA production capacity within the strain collection was conducted using TLC for qualitative detection [Figure 1E], identifying eight GABA-positive strains. Among them, DYDNL61M6 and DSCAB4M9 exhibited the strongest chromogenic signals. Further quantitative analysis of GABA levels in these 8 positive strains was carried out [Figure 1F], revealing that 6 were stable. DYDNL61M6 exhibited the highest GABA production (25.61 g/L), while DQHXNQ05M30 showed the lowest yield (5.34 g/L).

Based on phylogenetic and functional analyses, 3 representative strains were selected for subsequent mechanistic investigations: High GABA production (H-GABA): DYDNL61M6; Low GABA production (L-GABA): FSDHZD1L1; and No GABA production (NO-GABA): M2143. These strains exhibited significant differences in core genome sequences, key functional genes, and GABA production levels.

### Lactococcus lactis producing GABA alleviates IBS-like symptoms induced by transient Citrobacter rodentium infection combined with water avoidance stress in mice

**Figure 2:**
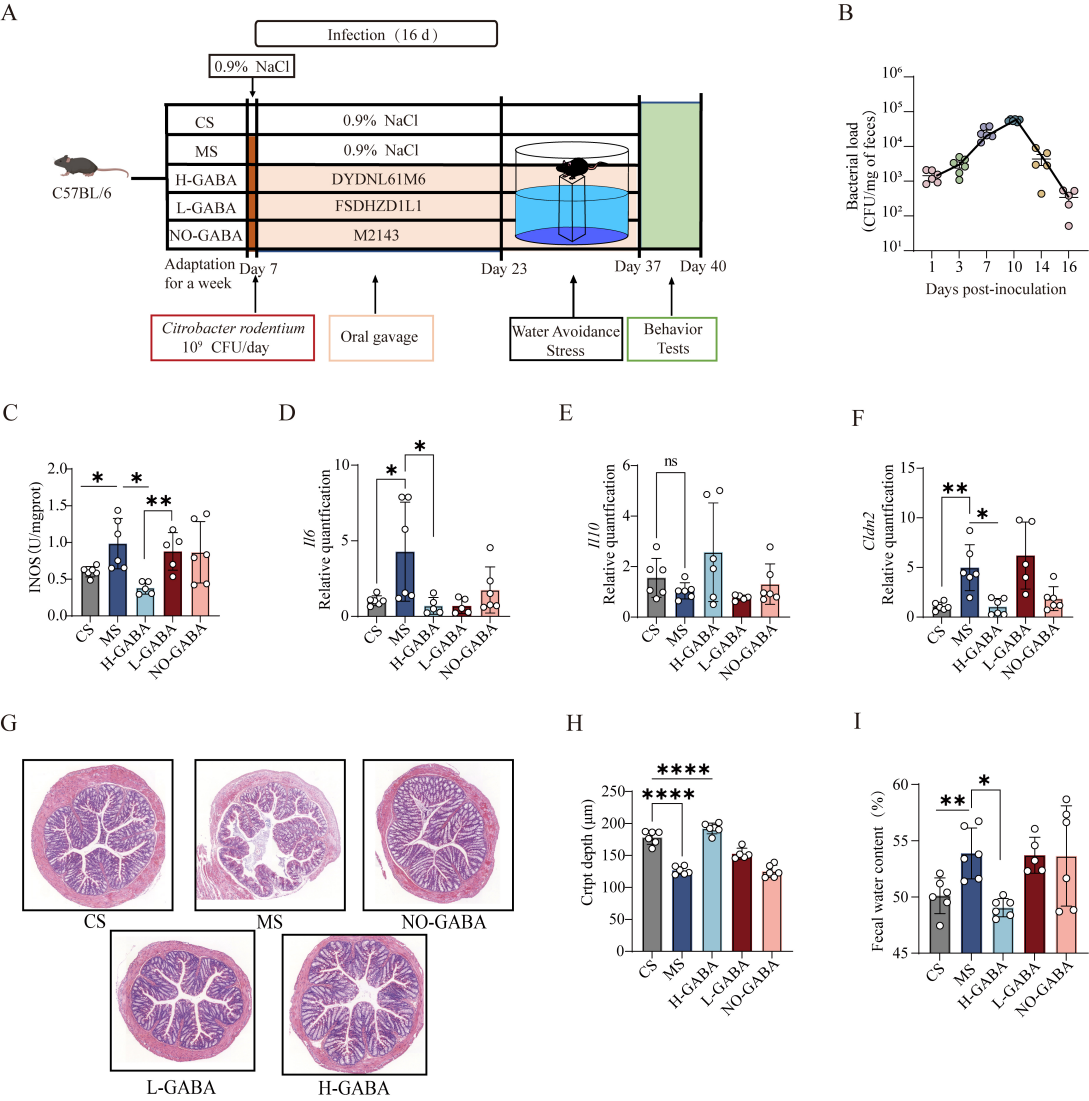
Alleviating effects of GABA-producing *Lactococcus lactis* on intestinal dysfunction in IBS model mice. (A) Schematic diagram of the experimental protocol for IBS model induction using *Citrobacter rodentium* infection combined with water avoidance stress; (B) CFUs of *Citrobacter rodentium* in feces of model mice at different time points after gavage; (C) Colonic iNOS enzymatic activity; (D-F) mRNA expression levels of inflammatory cytokines and barrier-associated proteins in colonic tissues: (D) *IL-6*, (E) *IL-10*, (F) *CLDN2*; (G) Representative H&E-stained colonic sections (10× magnification); (H) Quantification of colonic crypt length; (I) Measurement of fecal water content. Data were analyzed by one-way ANOVA followed by Dunnett’s multiple comparison test *vs.* the MS group. ns stands for *P* > 0.05, *P < 0.05; **P < 0.01; ***P < 0.001; ****P < 0.0001. GABA: Gamma-aminobutyric acid; IBS: irritable bowel syndrome; CFU: colony-forming unit; iNOS: inducible nitric oxide synthase; H&E: hematoxylin and eosin; MS: model + stress.

To mimic the clinical manifestations and stress-related pathophysiology of IBS, a murine model was established using *Citrobacter rodentium* infection in combination with water avoidance stress [Figure 2A]. From day 1 to 16 post-infection, the colonization of *Citrobacter rodentium* in the feces of the MS group exhibited a dynamic pattern of establishment, proliferation, stabilization, and clearance, indicating successful model induction [Figure 2B].

To evaluate the therapeutic potential and possible dose-dependency of GABA-producing *Lactococcus lactis* strains on IBS symptoms, mice were treated with H-GABA, L-GABA, or NO-GABA strains. Given that numerous studies have demonstrated a close association between NOS, particularly the iNOS, and visceral hypersensitivity in rodent models^[25-27]^, this study employed NOS activity as a surrogate indicator for visceral hypersensitivity mechanisms, aiming to reduce experimental variability while providing reliable mechanistic insights. The results showed that iNOS expression was significantly elevated in colonic tissues of both the MS and NO-GABA groups, suggesting the presence of visceral hypersensitivity (*P* < 0.05, Figure 2C). In contrast, treatment with the H-GABA significantly suppressed iNOS expression, while the L-GABA showed no significant effect, indicating a clear dose-dependent alleviation.

Given that IBS is often accompanied by mild inflammation and compromised intestinal barrier function, colonic expression levels of inflammation-related and barrier-related genes were further assessed. In the MS group, *IL-6* (*P* < 0.05, Figure 2D) expression was markedly upregulated, *IL-10* (*P* > 0.05, Figure 2E) expression showed a non-significant downward trend, and *CLDN2* (*P* < 0.05, Figure 2F) was significantly downregulated, collectively indicating inflammatory activation and impaired intestinal barrier function. Intervention with GABA-producing strains significantly reversed these alterations, with a mild trend of improvement also observed in the NO-GABA group. Histological findings supported these observations (*P* < 0.0001, Figure 2G and H): H&E staining revealed shallower colonic crypts and mild inflammatory cell infiltration in the MS group, whereas crypt architecture normalized and inflammatory infiltration decreased following H-GABA treatment. Moreover, mice in the MS and NO-GABA groups exhibited increased fecal water content, indicative of mild diarrhea. This was significantly reduced following H-GABA intervention (*P* < 0.01, Figure 2I), possibly due to improved intestinal permeability.

### GABA-producing Lactococcus lactis alleviates anxiety-like and cognitive impairment behaviors in IBS model mice

Patients with IBS often present with neuropsychological comorbidities such as anxiety, depression, and cognitive dysfunction. To assess the presence of similar neurobehavioral abnormalities in experimental mice, a battery of behavioral tests - including the OFT, NOR test, and FST - were employed.

**Figure 3:**
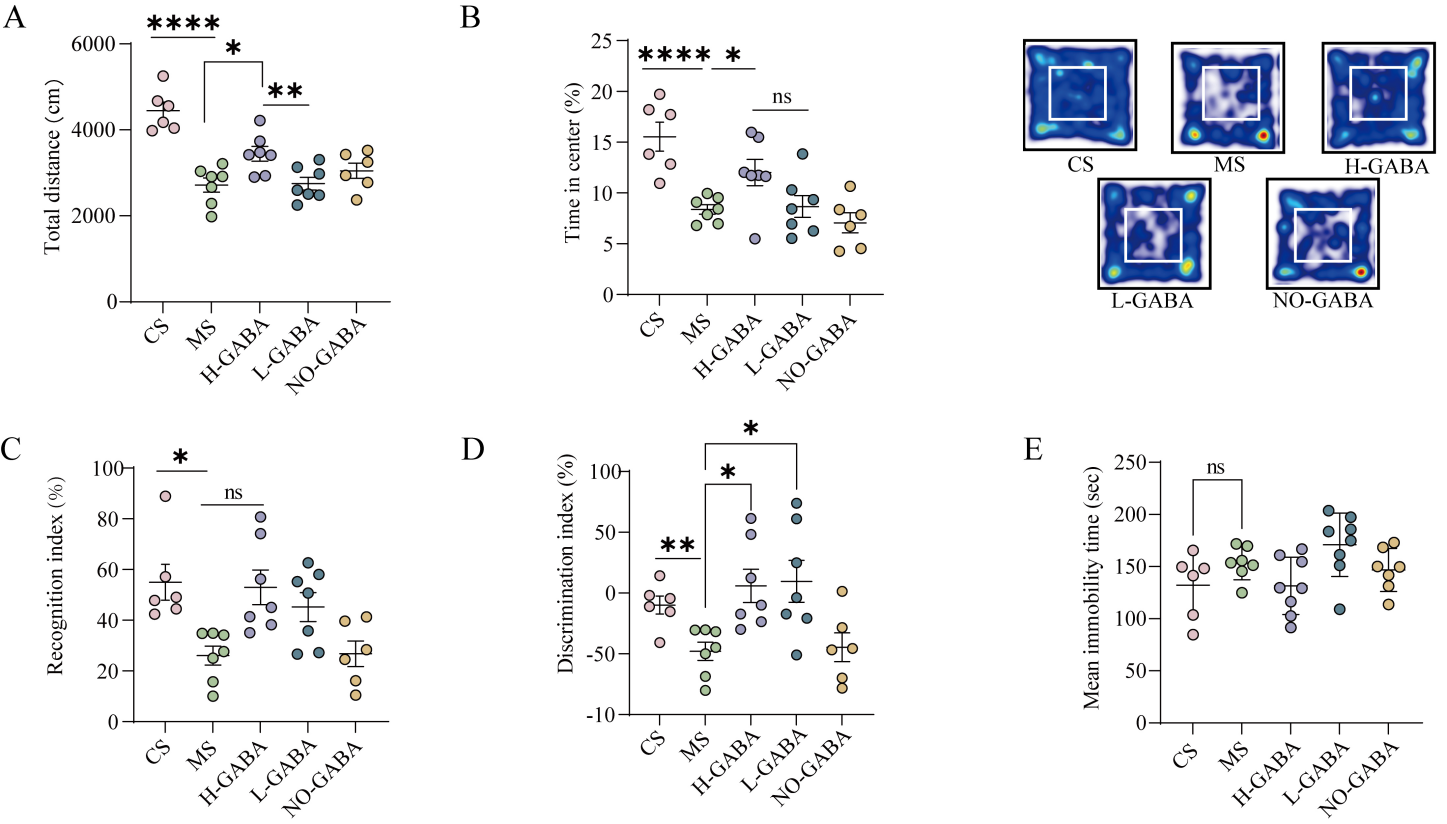
Effects of GABA-producing *Lactococcus lactis* on neurobehavioral abnormalities in IBS model mice. (A and B) OFT assessing anxiety-like behaviors: (A) Total distance, (B) Percentage of time spent in the center area and heatmaps of movement trajectories; (C and D) NOR test evaluating cognitive function: (C) Recognition index, (D) Discrimination index; (E) Immobility time in the FST used to assess depressive-like behavior. Data are presented as mean ± standard error of the mean (SEM). Statistical analysis was performed using one-way ANOVA followed by Dunnett’s multiple comparison test *vs.* the MS group. ns stands for *P* > 0.05, *P < 0.05; **P < 0.01; ***P < 0.001; ****P < 0.0001. GABA: Gamma-aminobutyric acid; IBS: irritable bowel syndrome; OFT: open field test; NOR: novel object recognition; FST: forced swim test; MS: model + stress.

In the OFT, mice in the MS and NO-GABA groups exhibited a significant reduction in both total distances traveled and frequency of entries into the central zone, with heatmaps showing activity predominantly restricted to the periphery (*P* < 0.0001, Figure 3A and B), indicating enhanced anxiety-like behavior. Both L-GABA and H-GABA interventions significantly ameliorated these anxiety-associated parameters, with the H-GABA group nearly reversing the anxious phenotype entirely, suggesting a potential dose-dependent effect.

In the NOR test, mice in the MS and NO-GABA groups displayed a marked reduction in both recognition (*P* < 0.05, Figure 3C) and discrimination indices (*P* < 0.01, Figure 3D), indicating mild cognitive impairment. Intervention with either L-GABA or H-GABA partially restored recognition ability; however, the intergroup differences did not show a clear dose-dependent trend.

To further assess potential depressive-like behaviors, the FST was conducted (*P* > 0.05, Figure 3E). No significant differences in immobility time were observed among the groups, suggesting the absence of overt depressive-like phenotypes. This observation aligns with clinical findings in IBS patients, where anxiety is more prevalent than depression.

### GABA-producing Lactococcus lactis alleviates IBS-related visceral hypersensitivity via modulation of GABA receptors and TRPV1

**Figure 4:**
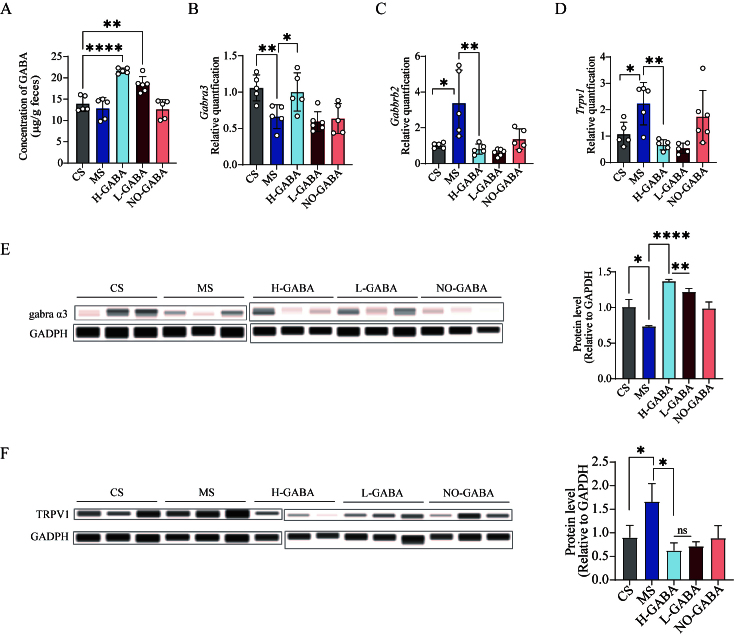
Regulatory effects of GABA-producing *Lactococcus lactis* on intestinal GABA signaling pathways. (A) GABA concentration in colonic contents; (B) mRNA expression of *GABRA13*; (C) mRNA expression of the *GABBR2*; (D) mRNA expression of TRPV1; (E) Protein expression of the GRBRA3; (F) Protein expression of TRPV1. Data were analyzed using one-way ANOVA followed by Dunnett’s multiple comparisons test against the MS group. ns stands for *P* > 0.05, *P < 0.05; **P < 0.01; ***P < 0.001; ****P < 0.0001. GABA: Gamma-aminobutyric acid; MS: model + stress.

Previous studies have demonstrated that elevated local levels of GABA exert anti-inflammatory effects by modulating the expression of GABA receptors, thereby alleviating intestinal inflammation^[14]^. To investigate this mechanism, we first quantified GABA concentrations in the colonic contents of mice (*P* < 0.0001, Figure 4A). The results showed that both L-GABA and H-GABA groups exhibited significantly higher GABA levels compared to the CS group, suggesting a dose-dependent relationship between bacterial GABA production capacity and luminal GABA concentrations.

Subsequently, we examined the expression of GABA-related receptors (*GABRA13, GABRA15, GABBR1, and GABBR2*) in colonic tissues. *GABRA13* was significantly downregulated in the MS and NO-GABA groups (*P* < 0.01, Figure 4B), while its expression was dose-dependently restored by GABA-producing strains. *GABBR2*, which encodes a GABA_B_ receptor subunit, was significantly upregulated in the MS group, whereas probiotic intervention markedly suppressed its expression (*P* < 0.05, Figure 4C). In addition, we further evaluated the expression of the visceral hypersensitivity marker TRPV1, which is closely associated with IBS pathophysiology and found that TRPV1 was significantly increased in the MS and NO-GABA groups (*P* < 0.05, Figure 4D). Finally, protein levels of GABBR2 and TRPV1 were analyzed to confirm these transcriptional changes, both of which exhibited consistent alterations (*P* < 0.05, Figure 4E and F).

### GABA-producing Lactococcus lactis modulates central nervous system activity via activation of GABA receptors

**Figure 5:**
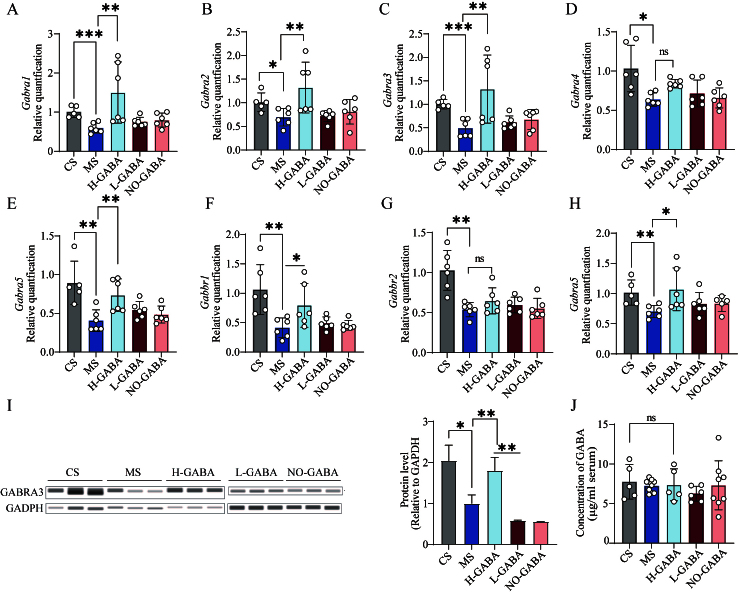
Regulatory effects of GABA-producing *Lactococcus lactis* on central GABA signaling pathways. (A-G) mRNA expression levels of GABA receptor-related genes in the hippocampus: (A) *GABRA11*; (B) *GABRA12*; (C) *GABRA13*; (D) *GABRA14*; (E) *GABRA15*; (F) *GABBR1*; (G) *GABBR2*; (H) mRNA expression of the *GABRA13* in the amygdala. (I) Protein expression of GABRA13 in the hippocampus; (J) GABA concentration in serum. Data were analyzed using one-way ANOVA followed by Dunnett’s multiple comparisons test against the MS group. ns stands for *P* > 0.05, *P < 0.05; **P < 0.01; ***P < 0.001. GABA: Gamma-aminobutyric acid; MS: model + stress.

To investigate whether GABA-producing *Lactococcus lactis* exerts anxiolytic effects through modulation of central GABAergic signaling, the expression of GABA_A_ and GABA_B_ receptor subunits in the hippocampus and amygdala was evaluated. In the hippocampus, mRNA expression levels of GABA_A_ receptor subunits (*GABRA11*, *GABRA12*, *GABRA13*, *GABRA14*, *GABRA15*) as well as GABA_B_ receptor subunits (*GABBR1* and *GABBR2*) were significantly downregulated in the MS group. (*P* < 0.05, Figure 5A-G). Intervention with the H-GABA significantly upregulated these genes, whereas the L-GABA induced a similar but less pronounced trend, suggesting a dose-dependent effect. In contrast, within the amygdala, only the mRNA expression of *GABRA15* was significantly reduced in the MS group. This reduction was restored to control levels following treatment with the H-GABA (*P* < 0.01, Figure 5H). No significant changes were observed in the expression of other GABA receptor subunits in the amygdala. Consistent with the mRNA, the protein levels of the corresponding hippocampal subunits were assessed, but no statistically significant changes were detected (*P* > 0.05, Figure 5I). Notably, serum GABA concentrations did not differ significantly among the experimental groups (*P* > 0.05, Figure 5J).

## DISCUSSION

In this study, we systematically evaluated the therapeutic effects of GABA-producing *Lactococcus lactis* in a mouse model of IBS and investigated its potential mechanisms. The results demonstrated that GABA-producing strains significantly ameliorated multiple pathological features, with several parameters exhibiting a clear dose-dependent response. Specifically, the intervention markedly improved visceral hypersensitivity, intestinal barrier dysfunction, and inflammatory responses in IBS model mice, while also mitigating neurobehavioral abnormalities such as anxiety and cognitive deficits. Moreover, administration of these strains significantly upregulated the expression of GABA receptors in both the gut and brain, suggesting that the probiotic effects may be mediated through modulation of GABAergic signaling along the gut-brain axis.

Visceral hypersensitivity is one of the most prominent and clinically significant symptoms of IBS, typically characterized by a lowered threshold for visceral distension and stimulation, leading to exacerbated symptoms such as abdominal pain and bloating. Previous studies have demonstrated that the sensitization of TRPV1 plays a critical role in the visceral hypersensitivity observed in IBS patients^[28]^. In mouse models with TRPV1 gene knockout or ablation of TRPV1-expressing neurons, IBS-induced visceral nociceptive responses are significantly attenuated^[29]^, further confirming the essential role of TRPV1 in sensory hypersensitization. The expression and activity of TRPV1 are modulated by various internal and external factors, including proinflammatory cytokines, alterations in neurotransmitters, and disruption of intestinal barrier integrity^[30]^. In the present study, we observed a significant upregulation of the proinflammatory cytokine *IL-6* and a downregulation of the tight junction protein *CLDN2* in the colonic tissue of MS group, accompanied by increased transcription of TRPV1 and the protein levels of TRPV1, indicating a sensitized state of local TRPV1 channels. TRPV1 sensitization enhances the responsiveness of intestinal sensory nerve endings to external stimuli and contributes to increased excitability of local neural circuits, forming the pathological basis of visceral hypersensitivity^[31]^. Conversely, GABA receptors - particularly the GABA_A_ subtype - serve an important inhibitory role in the enteric nervous system. By promoting Cl^-^ influx, GABA_A_ receptors induce neuronal hyperpolarization, thereby reducing aberrant neuronal firing and dampening hyperexcitability^[32]^. Recent studies have suggested that the α1 and α3 subunits of GABA_A_ receptors may act as key modulators of immune signaling and neurotransmission in inflammation-associated intestinal disorders^[33]^. Our findings further demonstrate that intervention with GABA-producing *Lactococcus lactis* significantly upregulated the expression of the GABRA3 and concurrently downregulated TRPV1 expression in a dose-dependent manner. Based on these observations, we propose that *Lactococcus lactis* strains capable of producing GABA may alleviate visceral hypersensitivity in IBS by enhancing local GABAergic signaling, thereby suppressing TRPV1-mediated neuronal sensitization and reducing the excitability of colonic afferent nerves.

Emotional disturbances, particularly anxiety, are among the most common comorbidities in patients with IBS, significantly impairing quality of life. Current evidence indicates that dysregulation between excitatory and inhibitory neurotransmission constitutes a key mechanism driving the pathogenesis of anxiety disorders^[34]^. The hippocampus, a key brain region involved in anxiety regulation, is densely populated with GABAergic interneurons, which play a central role in maintaining neuronal circuit stability and emotional homeostasis through inhibitory modulation. However, these neurons are highly susceptible to functional disruption under chronic stress and other external stimuli^[35,36]^. In the present study, we observed a marked downregulation of GABA receptor subunits in both the hippocampus and amygdala of MS group, suggesting weakened inhibitory GABAergic function and a possible state of neuronal hyperexcitability, particularly in the hippocampus. This alteration aligns with the emergence of anxiety-like behaviors associated with IBS. Notably, intervention with GABA-producing *Lactococcus lactis* strains effectively restored GABA receptor expression and significantly alleviated anxiety-like behaviors in mice, suggesting that these strains may exert neuromodulatory effects by enhancing central GABAergic signaling. Interestingly, serum GABA levels did not differ significantly among groups, consistent with previous findings that GABA, due to its high polarity, poorly penetrates the blood-brain barrier^[37]^. This suggests that the observed effects are unlikely to be mediated by GABA transport through the gut-blood-brain systemic circulation. In contrast, the vagus nerve, as a central conduit of gut-brain axis communication, is widely recognized as a critical hub through which peripheral signals reach the central nervous system^[38-40]^. Probiotic studies illustrate this role: *Lactobacillus rhamnosus JB-1* reduced anxiety- and depression-like behaviors in mice, effects abolished by subdiaphragmatic vagotomy^[41]^. Similarly, fecal microbiota transplantation from dysbiotic mice induced neuroinflammation and depression-like phenotypes, which were prevented by vagotomy^[42]^. Inflammatory signaling further underscores vagal importance. Vagotomy exacerbates DSS-induced colitis and elevates colonic proinflammatory cytokines^[43]^, whereas an intact vagus mediates anti-inflammatory effects, as shown by fluoxetine’s attenuation of LPS-induced cytokine responses, which is lost after vagotomy^[44]^. Notably, the anxiolytic effects of exogenous GABA also depend on vagal integrity; vagotomy diminishes both intestinal and behavioral responses, while GABA supplementation mimics vagotomy’s effects, suggesting the vagus mediates GABA-driven gut-to-brain signaling^[45]^. Building on this evidence, we speculate that GABA-producing strains may increase colonic GABA levels, activate gastrointestinal vagal afferents, and subsequently modulate GABA receptor expression in the hippocampus and amygdala, ultimately alleviating IBS-associated anxiety-like behaviors.

This study systematically evaluated the effects of GABA-producing *Lactococcus lactis* on alleviating intestinal dysfunction and neurobehavioral abnormalities in an IBS-D mouse model, and explored its potential regulatory mechanisms within the gut0brain axis. However, several limitations remain. First, direct evidence is still lacking regarding the colonization stability of the administered strains in the colon and the *in vivo* dynamics of GABA production. Although *Lactococcus lactis* is generally thought to synthesize GABA via the glutamate decarboxylase (GAD) pathway^[46]^, the efficiency of this process and its regulation by substrate availability *in vivo* have not been fully elucidated. Our measurements were performed under substrate-rich conditions to reflect strain-specific conversion capacity, rather than substrate utilization limits. Future studies incorporating colonic GABA quantification, metabolic kinetics, and isotope tracing would provide more robust insights into how these strains regulate GABA levels *in vivo* and influence gut-brain communication. Second, the NO-GABA used in this study may retain residual bioactivity, making it an imperfect negative control. Future studies should aim to construct GABA synthesis-deficient knockout strains to enable more precise functional validation. Finally, combining this approach with vagotomy experiments would help clarify whether the central regulatory effects of GABA signaling depend on vagal transmission, thereby enhancing the rigor and interpretability of mechanistic inferences.

In summary, GABA-producing *Lactococcus lactis* alleviates both intestinal dysfunction and neurobehavioral disturbances in IBS model mice by modulating GABAergic signaling within the gut-brain axis. These findings provide experimental evidence and theoretical support for the development of probiotic interventions targeting GABA metabolism.

## DECLARATIONS

### Availability of data and materials

All data are available in the main text. Other raw data can be available from the corresponding author upon reasonable request.

### Copyright

© The Author(s) 2025.
